# Membranes Based on Cellulose and Copolymers of Acrylonitrile Prepared from Joint Solutions

**DOI:** 10.3390/membranes13070667

**Published:** 2023-07-14

**Authors:** Igor S. Makarov, Gulbarshin K. Shambilova, Markel I. Vinogradov, Tatyana S. Anokhina, Aigul S. Bukanova, Fazilat B. Kairliyeva, Saule K. Bukanova, Ivan S. Levin

**Affiliations:** 1 A.V. Topchiev Institute of Petrochemical Synthesis, Russian Academy of Sciences, Leninsky Prospect, 29, 119991 Moscow, Russia; m.i.vinogradov1989@yandex.ru (M.I.V.); tsanokhina@ips.ac.ru (T.S.A.); levin@ips.ac.ru (I.S.L.); 2 Institute of Petrochemical Engineering and Ecology named after N.K. Nadirov, Atyrau Oil and Gas University named after S. Utebayev, Baimukhanov Street, 45A, Atyrau 060027, Kazakhstan; shambilova_gulba@mail.ru (G.K.S.); bukanova66@mail.ru (A.S.B.); kairlieva.fazi@mail.ru (F.B.K.); sauleshik81@mail.ru (S.K.B.); 3 Department of Chemistry and Chemical Technology, Kh. Dosmukhamedov Atyrau University, Studenchesky Ave., 1, Atyrau 060011, Kazakhstan

**Keywords:** composite membranes, nanofiltration, copolymers of acrylonitrile, N-methylmorpholine-N-oxide, morphology, rheology, structure

## Abstract

Cellulose and copolymers of acrylonitrile (PAN) are characterized by their chemical resistance to several conventional solvents. Therefore, these polymers are often used to obtain membranes for the recovery of such solvents. In this work, for the first time, composite membranes formed from highly concentrated mixed solutions based on cellulose and PAN are considered (the total content of polymers is 18 wt.%). For mixed solutions, the morphology and rheological behavior were evaluated. It is shown that the resulting solutions are two-phase, and their morphology depends on the components’ ratio and the system’s history. The non-monotonous change in the viscosity with the PAN content indicates a specific interaction of cellulose and PAN in N-methylmorpholine-N-oxide solutions. The rheological behavior of mixed solutions allows for their processing in conditions identical to those of cellulose solutions. The introduction of PAN into the cellulose matrix promotes a decrease in the structural order in the system, affecting the membranes’ transport properties. For composite membranes, it was found that with an increase in the content of the PAN phase, the retention of Remazol and Orange decreases, while the observed values are several times higher than those for cellulose membranes. The permeability of ethanol increases with increasing terpolymer content.

## 1. Introduction

The excellent properties of cellulose (chirality, hydrophilicity, degradability, chemical variability, high tensile strength, etc.) have determined its dominance in different applications for many years. Practically inexhaustible and constantly renewable raw materials, besides the traditional construction industry, are found in more sophisticated areas of application, such as artificial fibers, films, microcrystalline powder fillers, and green biomass, etc., which may be referred to as high-tech products [1,2]. Unfortunately, the structural organization of the hydrogen bond system excludes the transformation of cellulose into a flow state by increasing the temperature, i.e., the destruction of cellulose starts at temperatures much lower than its melting point (467 °C) [3]. Leaving aside the chemical modification of cellulose, let us consider the processing of cellulose via solutions. The number of direct cellulose solvents is limited because of the high rigidity of macromolecules connected by strong H-bonds [4,5]. An active search for cellulose solvents led to the discovery of a set of dissolving systems (cuprammonium, LiCl/N,N-dimethylacetamide (DMAc), ionic liquid, NaOH/urea, N,N-dimethylformamide (DMF)/N_2_O_4_, orthophosphoric acid, etc.), but unfortunately, their practical application is limited due to either high cost, corrosive activity, difficulties of regeneration, or toxicity. The most promising solvent is N-methylmorpholine-N-oxide (NMMO), which is used in the so-called MMO process [6]. The production of cellulose fibers from solutions in NMMO reaches hundreds of thousands of tons, while films from these solutions are not produced on an industrial scale. The main type of cellulose film remains cellophane, which, like fibers, is produced using an ecologically dangerous viscose process. This circumstance leaves open the question of finding the optimal conditions and regimes for the production of cellulose films, for example, as barrier films.

Cellulose-based films have been engaged in their special niche among membrane materials. Membranes from cellulose and its derivatives are known for their mechanical properties, hydrophilicity, and resistance to a large number of organic solvents, including petroleum products, hypoallergenic properties, biodegradability, and so on. In addition, the structural features of cellulose render it possible to use this material for the separation of liquid (binary mixtures of glycerol, ethylene glycol, ethanol, formalin, acetone, and water) and gaseous (CO_2_/H_2_) mixtures. The biocompatibility of cellulose allows for the use of such membranes in medicine, for example, dialysis membranes, supercapacitor membrane separators, drug capsules, and cellulose scaffolds, etc. [7].

Abe et al. [8,9] were among the first to apply the cellulose films obtained via the NMMO process as membranes for hemodialysis, along with well-known membrane materials such as cellophane and cuprofan [10]. It is noted in [11] that the structure of membranes formed by viscose and MMO processes is fundamentally different. Membranes prepared from solutions in NMMO have higher tensile strength (up to 75 MPa) and modulus (up to 2.5 GPa), whereas cellophane films have higher deformability and elongation at break (65%) [11]. The authors of [12] proposed the use of films obtained from cellulose solutions in NMMO as membranes with better separation performance concerning water-alcohol media.

While having several advantages, cellulose membranes demonstrate low separation rates (permeation coefficients) and they remain virtually impervious to methanol, ethanol, and acetone, etc. [13]. This phenomenon is not affected by the reduction in thickness of the film. The way out of this situation was found in the 1930s by employing the preliminary water activation of the cellulose film with the subsequent replacement of water by a less polar liquid (ethanol, methanol, etc.) [14]. Recently, Yushkin et al. [15] have shown that the activation of cellulose film by its treatment with media of lower polarity makes it possible to use cellophane as a nanofiltration membrane or for the separation of organic solvents.

It would be reasonable to assume that such activations of cellulose films are accompanied by structural changes. This means [16] that not only the composition of the coagulant influences the structure and properties of the prepared membranes, but also the subsequent washing of the gel-like films to completely remove the solvent from the system, which leads to significant changes in the structure of the resulting membranes.

In respect to the morphology of the finished cellulose films formed from solutions in NMMO, there is no unanimous agreement regarding the results obtained. A smooth film surface without visible pores was described in [17,18], while a rough surface with an average pore size of 7.7 nm and 50–179 nm was found in [9,19]. Such discrepancy in the results can be caused both by the conditions of film preparation and subsequent drying, which introduces a significant contribution to features of the final structure. Based on the example of Lyocell fibers, the evolution of the structure under swelling and dehydration conditions was traced [20,21,22,23,24]. The average pore size when removing the water increases by several times and the total number of pores is reduced by “collapsing” part of the pores. Subsequent drying and wetting of the fibers also led to an increase in the orientation order in the system.

Thus, an increase in the permeate rate through the membrane can be achieved by the creation of a suitable structure and morphology of the films, which is achieved in the process of membrane preparation by controlling the conditions of film formation, namely the temperature-concentration parameters of the dopes, composition and temperature of the coagulant, and drying conditions, etc.

Along with the aforesaid methods of changing the structure of cellulose, it is possible to regulate it using additives to the dope. The solid-phase method of “dissolving” the cellulose in NMMO [25] allows us to perform this operation at an initial stage, i.e., in the course of mixing and activating the cellulose–additive–solvent system [26]. Utilization as an additive to cellulose of polymers, for example, PAN, which is often used for producing micro- and ultrafiltration membranes, can help reach high selectivity. In addition, PAN is also known for its high resistance to a large quantity of solvent, which will allow not only variation to the structure of membranes but also influence of their mechanical and transport characteristics.

In previous works, the preparation of polyacrylonitrile (PAN) solutions [27,28] and joint cellulose-PAN solutions in NMMO, as well as the composite fibers [29], was demonstrated. The introduction of up to 5 wt.% of PAN into the cellulose matrix did not significantly affect the rheological behavior of the mixed solutions but allowed for changing the mechanical and thermal properties of fibers [30,31].

In the present work, the main tasks are the following:

- to obtain the highly concentrated solutions in NMMO with various ratios between cellulose and PAN,
- to study the rheological behavior of the mixed solutions,
- to form composite membranes from these dopes and to evaluate their structure, morphology, mechanical, and transport properties.

## 2. Materials and Methods

### 2.1. Materials

Cellulose powder with DP = 600, moisture ~8 wt.%, and alpha-cellulose content not less than 92 wt.%) was supplied by Baikal Pulp and Paper Mill (Baykalsk, Russia). PAN copolymer as a powder with an average particle size of 50 μm (93.9 wt.% acrylonitrile, 5.8 wt.% methyl acrylate, and 0.3 wt.% sulfonic acid methyl ester, with a molecular weight of 85 kg/mol) was provided by Goodfellow (Huntingdon, UK). The direct solvent of cellulose N-methylmorpholine-N-oxide (NMMO) (T_m.p._ = 110–130 °C, H_2_O < 10 wt.%) was purchased from Demochem (Shanghai, China), and Propylgallate was supplied by Sigma-Aldrich (St. Louis, MO, USA). N-decane (Component-Reactive, Moscow, Russia) with a density of 0.735 g/cm^3^ was used to measure the sorption capacity of cellulose membranes. The ethanol used in this work contained 4 wt.% of water and was not further desiccated. Negatively charged dyes Remazol Brilliant Blue R (MW = 626 g·mol^−1^) and Orange II (MW = 350 g·mol^−1^) were used as model substances. All reagents were chemically pure and used without additional purification.

### 2.2. Preparation of Mixed Solutions

All the above-mentioned chemicals were used as the main components for the 18 wt.% mixed solution preparation. Based on the previously developed process for solid phase dissolution of cellulose in NMMO [32], the method has been developed for the preparation of mixed solutions containing up to 50 wt.% PAN, which includes three main stages: mechanical mixing of components, solid phase activation under compression and shear deformation, and transformation in a fluid state of the system by heating to the melting temperature of the solvent. Heating of the activated cellulose-PAN-NMMO system and production of the mixed solution were performed in the working unit of the HAAKE Minilab II twin-screw mixer (Karlsruhe, Germany), and the rotation speed of the screws was 100 rpm, temperature of 120 °C. The preparation time of the dope did not exceed 2–5 min.

The quality of the solutions obtained, and their morphology, were studied by polarizing microscopy by Boetius (VEB Kombinat Nadema GmbH, former DDR).

### 2.3. Preparation of the Membranes

Mixed solutions obtained on a HAAKE Minilab II twin-screw mixer were used to form membranes after evaluating their quality. To obtain membranes, a laboratory setup for forming thin films, the HLCL-1000 laminator (Cheminstruments), was used. The membranes were formed at a temperature of 120 °C, and the temperature control of the solution was performed for 3–5 min. The thickness of the future membranes was controlled by varying the height of the gap between the shafts. For this, calibration plates of a given thickness were used. To exclude the adhesion of the mixed solution to the shafts, two cover films made of polyimide and polyethylene terephthalate were used. After the formation of the membrane, the upper substrate was immediately removed. After that, the sample on the bottom substrate was immersed in an aqueous coagulation bath (T = 20 ± 2 °C). The volume of the bath was 1.0 L. After contact with water, the processes of solvent removal and polymer phase separation were initiated, which made it possible to remove the second substrate without any extra effort. After the precipitation bath, the membranes were additionally washed with distilled water until the solvent was completely removed.

### 2.4. Characterization Methods

#### 2.4.1. Rheology

The rheological properties of the mixed solutions in NMMO were studied on a rotational rheometer Kinexus pro+ («Malvern Panalytical», Malvern, UK) with a 20 mm cone-plate operating unit (the angle between the element of the cone and the plane was 1°). The tests were conducted under steady-state conditions in a controlled shear rate regime. The speed range was 0.01–10 s^−1^ at a temperature of 120 °C. Dynamic tests were conducted in a regime corresponding to the linear viscoelasticity range in the frequency range 0.1–100 Hz with a constant deformation of 5%. Each rheological curve was always obtained 3–5 times to check the data reproducibility.

#### 2.4.2. Mechanical Properties of Wet Membranes

The mechanical properties of the wet cellulose and composite membranes were evaluated on an Instron 1122 tensile testing machine (Instron, Norwood, MA, USA) with a stretching rate of 10 mm/min and a base between pneumatic clamps of 10 mm. Samples for mechanical testing were cut using a 4 cm by 1 cm sharp-edged frame. The tests were performed at a temperature of T = 22 ± 1 °C. The film thickness was measured with an electronic micrometer. To avoid deformation of the films by the measuring cylinders of the micrometer, they were preliminarily placed between two coverslips. Twenty tests for each group of samples were performed.

#### 2.4.3. Morphology of Composite Membranes

The study of the morphology of cellulose and composite membranes was conducted on dry samples. The morphology of the membranes’ transverse cleavages was investigated by low-voltage scanning electron microscopy (LVSEM) on an FEI Scios microscope (Waltham, MA, USA) at an accelerating voltage of less than 1 kV in the secondary electron mode [33]. Cleavages were formed after freezing in liquid nitrogen perpendicular to the plane of the sample.

#### 2.4.4. Structure of Composite Membranes

The structure of the cellulose and composite membranes was studied by X-ray diffractometry on a Rigaku Rotaflex D/MAX-RC setup equipped with a rotating copper anode (X-ray source operating mode 30 kV, 100 mA, characteristic radiation wavelength λ = 1.542 Å, CuKβ radiation absorbed by a nickel filter), a horizontal goniometer, and a scintillation detector. X-ray experiments are performed in the reflection mode according to the Bragg–Brentano scheme in the continuous θ–2θ scanning mode in the angular range of 3°–40.0° with a scanning step of 0.04° at 20–22 °C.

#### 2.4.5. Sorption of N-Decane

The available volume in the investigated membranes was determined through the adsorption of an inert liquid (n-decane) by films according to the procedure described in [34]. Every sorption measurement was a triple determination, and the average values are reported.

#### 2.4.6. Flux and Nanofiltration Characteristics of the Membranes

**Figure 1:**
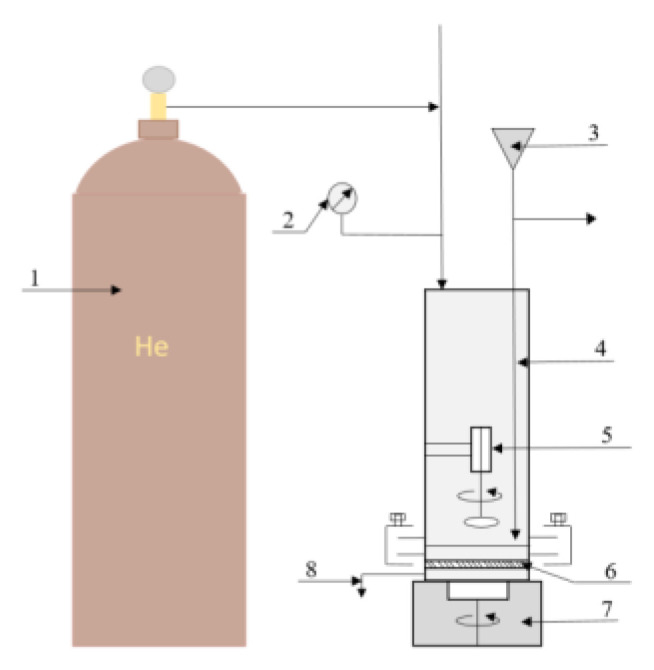
Schematic diagram of the filtration cell: 1—gas cylinder (helium), 2—manometer, 3—liquid supply system to the cell, 4—filtration cell, 5—magnetic anchor, 6—membrane, 7—magnetic stirrer, 8—sampler.

The study of ethanol permeability of cellulose membranes modified by polyacrylonitrile was conducted in a dead-end filtration cell (Figure 1) with a transmembrane pressure of 10 atm equipped with a magnetic stirrer by applying the procedure described above [35]. The installation is completely made of stainless steel, and rubber rings were used as seals. The active area of the membrane in the cells was 16.6 cm^2^. The volume of liquid was chosen in such a way that no more than 20% of the solution passed through the membrane during the experiment. The pressure in the cell was created with helium. Before the experiment, the membrane samples were not subjected to water removal but were kept in water.

The flux of permeate was determined by the weight method. For this purpose, a liquid receiver was installed at the outlet of the cell, designed in such a way as to minimize the evaporation of the permeate during the accumulation of the fluid sample. Measurement of the permeate mass of the permeate passed through the membrane during the experiment was conducted using the laboratory balance of the firm “Sartorius” with a measurement error of 0.001 g. The capacity of the membrane was characterized by liquid permeability (*P*), which was calculated as follows:

(1) $$P=\frac{m}{S\cdot \Delta t\cdot \Delta p}$$

where *m* is the mass (kg) of the permeate passed through the membrane with area *S* (m^2^) over a time Δ*t* (h) at a pressure differential Δ*p*.

At least five membranes of each type were tested to calculate the average values of the permeability.

## 3. Results and Discussion

**Figure 2:**
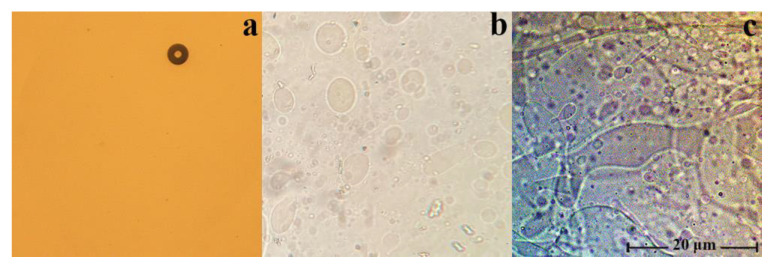
Micrographs of 18 wt.% solutions cellulose with PAN in NMMO, the ratio of polymers in solution: 100:0 (**a**), 90:10 (**b**), and 80:20 wt.% (**c**).

The morphology of the mixed solutions of cellulose with PAN in NMMO was studied with polarized optical microscopy, and it was found that in the whole range of concentrations, they are emulsions of different morphology (Figure 2).

**Figure 3:**
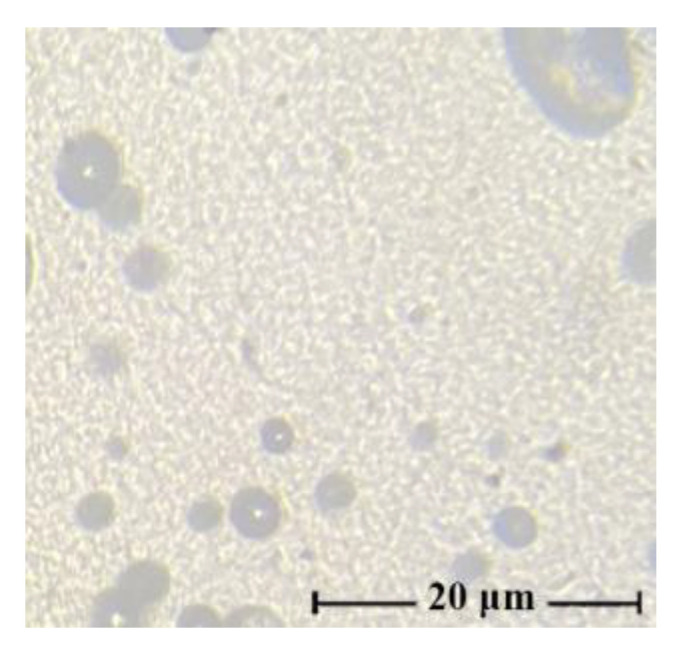
Micrographs of 18 wt.% solutions cellulose with PAN in NMMO after intensive mixing, the ratio of polymers in solution: 60:40 wt.%.

In such cases (as can be seen from the figure), the morphology of the mixture solutions varies from the drop solution, with an average droplet size of several microns for systems with low additive content (up to 10 wt.%), to randomly distributed extended structures (dispersed phase), with a PAN concentration in solution of more than 20 wt.%. With intensive mixing of a two-phase solution, the morphology of the system changes dramatically. Depending on the content of PAN in the solution, it varies from a fine droplet to an interpenetrating morphology. Figure 3 shows a micrograph of a solution containing 40 wt.% PAN.

For the resulting system (60 wt.% cellulose–40 wt.% PAN), it is difficult to identify the dispersion medium and dispersed phase. Thus, the morphology of mixed solutions depends not only on the ratio of components but also on the history of the obtaining system (temperature, intensity, and time of mixing).

**Figure 4:**
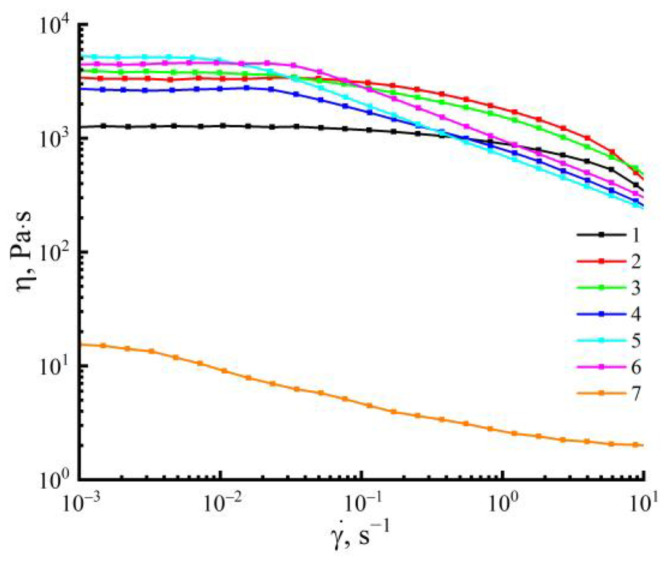
Flow curves of 18 wt.% solutions of cellulose (1), PAN (7), and mixed solutions of cellulose-containing 10 (2), 20 (3), 30 (4), 40 (5), 50 wt.% (6) PAN in NMMO at 120 °C.

Solutions of cellulose with PAN in NMMO constitute stable, biphasic emulsions, where a lower-viscosity solution with PAN in NMMO is contained in a high-viscosity cellulose solution in NMMO. When the concentration of PAN in the system increases (and, accordingly, the content of cellulose decreases), the values of the viscosities of coexisting solutions get closer to each other. Changing the character of the emulsion is reflected in the rheological behavior of the mixed solution (Figure 4).

The viscosities of the solutions of the initial components in NMMO differ significantly. The PAN solution in NMMO has a viscosity that is almost three times less than the cellulose solution. For solutions containing up to 10 wt.% of the PAN (drop emulsion), the character of the flow curve is similar to that of pure cellulose, and the Newtonian behavior remains up to 1 s^−1^. At higher shear rates, the viscosity decreases, and the non-Newtonian nature of the flow is observed. In cases where the PAN content in the system is up to 20–40 wt.% (an emulsion with a random distribution of extended dispersed phase structures), there is a decrease in the portion of the Newtonian flow. Transition to the non-Newtonian behavior of the flow is observed at lower shear rates. With an increase in the PAN content to 40 wt.% in the Newtonian flow region, the viscosity changes insignificantly. At 50 wt.% of the PAN content, almost no region of the Newtonian flow is observed.

**Figure 5:**
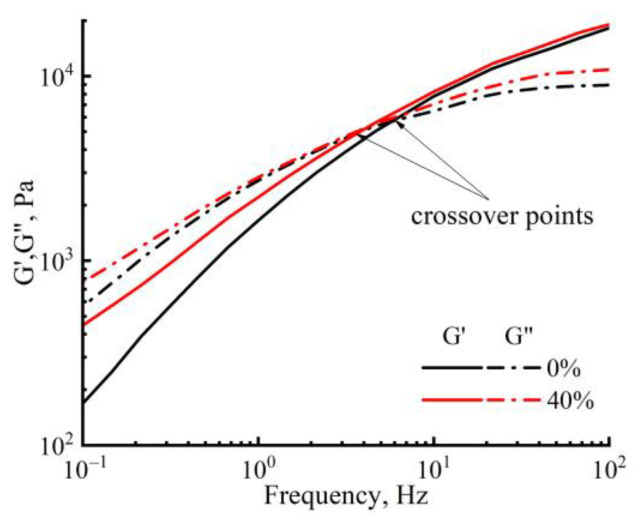
Frequency dependences of dynamic moduli of cellulose and a mixed solution of cellulose with PAN at 120 °C.

In Figure 5 the dependence of the modulus of elasticity and the loss modulus on the oscillation frequency at a temperature of 120 °C (for cellulose solutions with different PAN content), an increase can be observed in the values of both moduli with increasing frequency. The introduction of PAN in small quantities does not affect the viscoelastic properties, but with an increase in its concentration up to 40 wt.%, the shift of the crossover to the lower frequencies takes place. This means an increase in the elastic response of the mixed solutions compared with the cellulose solution. Therefore, the rheological behavior of mixed solutions is determined by their prehistory (morphology) and temperature.

**Figure 6:**
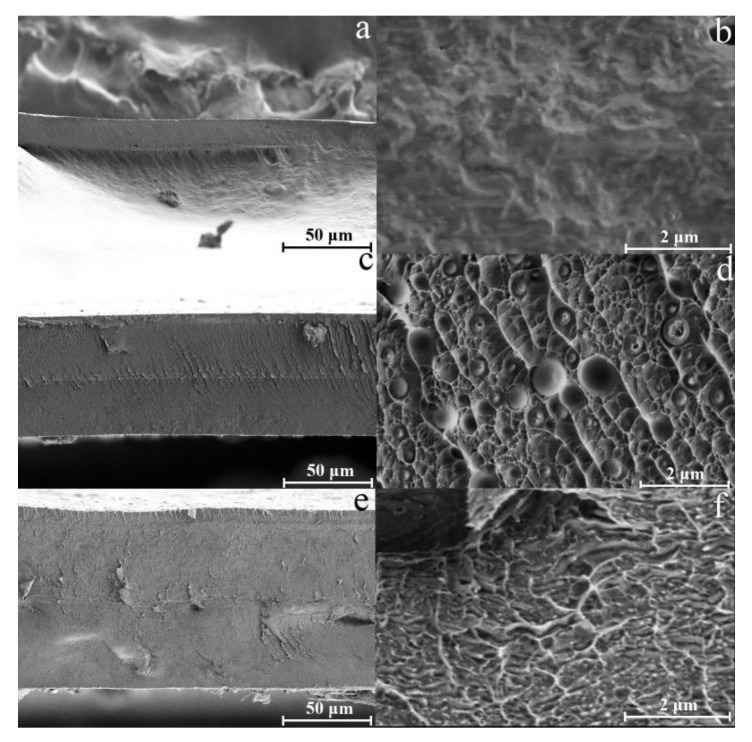
SEM-micrographs of the membranes cross sections: cellulose (**a**,**b**), composite membranes, containing 90 wt.% cellulose and 10 wt.% PAN (**c**,**d**) and 80% cellulose and 20 wt.% PAN (**e**,**f**).

Relying on the data of rheological research, cellulose and composite films with an additive content of up to 50 wt.% of PAN were formed by calendering. The morphology of the composite membranes is shown in Figure 6.

The morphology of cellulose membranes is characterized by a smooth surface and homogeneous texture without any obvious defects [11]. Regarding composite membranes, the one shown in Figure 6c,e has a morphological picture that differs slightly from cellulose membranes. The initially observed two-phase morphological pattern is reflected in the dispersed phase’s distribution over the membranes’ cross-section. For composite membranes with a PAN fraction of 10 wt.%, a morphology is observed consisting of a monolithic dispersion medium and spherical particles of the dispersed phase. The average particle size of the dispersed phase does not exceed 1 μm. With an increase in the PAN content in the system, small spheres are transformed into more extended formations and, upon separation from the solution during precipitation, form a monolithic structure with hard-to-identify phases. Although cellulose and PAN are radically different in nature, for membranes containing 20 wt.% PAN, phase separation is not observed in the micrographs.

**Figure 7:**
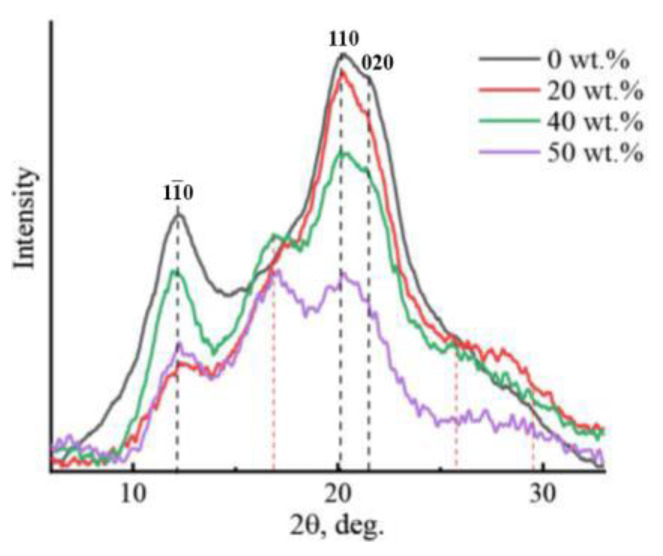
X-ray diffraction patterns of cellulose and composite membranes based on cellulose and PAN of different compositions.

Investigation of the structure of membranes by X-ray diffraction analysis made it possible not only to characterize the structure of cellulose and composite membranes but also to trace their transformation with increasing PAN concentration in the system (Figure 7).

As can be seen from Figure 7, for the obtained cellulose films, the main reflexes (marked in Figure 7) are in the typical cellulose allomorph II position (2θ~12.1°, ~20.1°, and ~21.5°) (planes 1–10, 110, and 020) [11,36]. For PAN, the diffraction patterns show reflections in the regions 2θ = 16.9°, 2θ = 29.4°, and a broad reflection in the region 25.7° [28], marked with a red dotted line. Interestingly, the introduction of PAN into cellulose in an amount of up to 5 wt.% does not lead to a significant change in the form of diffraction patterns; only reflection characteristics of cellulose are observed on them [30]. The behavior of the curves for composite membranes with a PAN content of up to 20 wt.% is similar. With a further increase in the PAN content in the system above 20 wt.%, characteristic PAN reflections begin to appear in the diffraction patterns. Inclusion into the cellulose matrix of PAN does not entail changes in the angular positions of the main cellulose reflexes. The intensity of the reflexes decreases with an increase in the concentration of the terpolymer in the system. On the contrary, the intensity of the characteristic PAN reflex in the region 2θ~16.9° (marked in Figure 7 with a red dotted line) increases, reaching its maximum values with an equal ratio of the components. Thus, a decrease in the concentration of cellulose on one side and an increase in the proportion of PAN in the system on the other leads to a decrease in the degree of ordering of the cellulose matrix. For the obtained diffraction patterns of composite membranes, the interlayer distance does not change, which allows us to speak about the degree of interaction between cellulose and PAN.

The reduction in structural organization in the system naturally affects the mechanical properties of composite membranes (Table 1).

As a rule, the mechanical properties of the membranes are investigated in dry conditions, but the utilization of cellulose membranes requires their preliminary aqueous activation. The change in the mechanical characteristics of the membranes in wet conditions will be investigated. The introduction of 10 wt.% PAN into cellulose results in a decrease in strength and elongation values. Probably, such a quantity of the additive does not allow it to be distributed evenly in the volume of the membrane during the forming process due to the high viscosity of the matrix solution and PAN particles behave as defects. A further increase in PAN in the system (up to 30 wt.% additive content) allows the achievement of better distribution in cellulose; however, the degree of cellulose ordering is reduced, which leads to a further decrease in the strength of the membranes. Membranes with a PAN content of 50 wt.% lose strength by up to 40% compared to samples without an additive. The relative elongation for membranes containing 10 wt.% PAN and more decreases monotonically in the case of an increase in the dispersed phase concentration. Membranes obtained from solutions with an equal content of cellulose and PAN have the same deformation properties as membranes made only of PAN [37].

Thus, the introduction of 10 and 20 wt.% PAN into cellulose leads to a decrease in strength from 3.9 MPa to 3.5 MPa, and a further increase in the PAN content to 50 wt.% reduces the membrane strength to 1.8 MPa. The elongation at break also decreases from 190% for cellulose membranes to 39% for composite membranes with a PAN content of 50%.

The characteristics of the membranes were investigated in terms of their ethanol permeability and free volume, by the adsorption of an inert liquid method. For all compositions, there were typical dependencies on the permeability of ethanol through a cellulose membrane modified with PAN. The experiments in respect of all cellulose and composite membranes were conducted after the values reached the stationary regime. The obtained values of ethanol permeability for cellulose films with different PAN contents are presented in Table 2.

As in the case of mechanical properties, a small number of PAN results in unusual properties of the membranes, namely, an increase in the permeability of composite membranes is observed. A further increase in the concentration of PAN leads to an increase in ethanol permeability. The maximum permeability of ethanol constitutes 6 kg/m^2^·h·atm which corresponds to the composite membrane with a PAN concentration of 50 wt.%. The values of the free volume, estimated by adsorption with n-Decane membranes, increase with the increase in PAN concentration. It can be assumed that this increase in ethanol permeability is due to the formation of additional porous PAN structures in the structure of composite membranes that are formed during the coagulation in water during the course of manufacture, which is confirmed by morphological and structural studies of such systems. It is known that PAN is widely used as a membrane-forming polymer for ultrafiltration membranes, as its pore size can reach 100 nm [38,39]. Such membranes are obtained by phase inversion, precipitating the formed membrane from the forming solution in the non-solvent (precipitant). A similar method was used in the present work for the production of cellulose membranes with PAN. It was shown in [11,37] that cellulose membranes, in contrast to PAN-based membranes, have low permeability and rejection of the Orange II dye. The rejection of the Orange II dye for cellulose membranes does not exceed 8%, while for PAN membranes, the rejection of Orange II is 74%, and for Remazol 97%.

**Figure 8:**
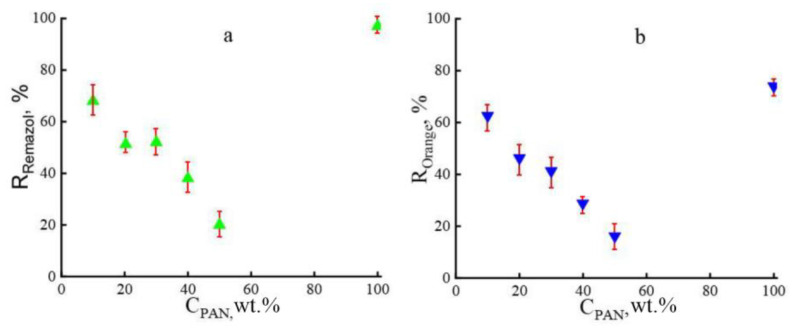
Concentration dependencies of the rejection for the dyes Remazol (**a**) and Orange II (**b**).

The separating properties of composite membranes were evaluated by the permeability of ethanol and its solutions with dyes, and the rejection was studied for the anionic dyes Remazol Brilliant Blue R and Orange II (Figure 8). For all systems, the values were estimated after the end of the period of initial relaxation of the membrane and reaching the steady-state flow regime.

In contrast to cellulose membranes, composite membranes, regardless of the PAN content in the system, showed the best separating characteristics when the model substances were removed from ethanol. The Orange II rejection decreases with increasing PAN content in the system by up to 50 wt.%. However, even with such a strong decrease in Orange II rejection, the observed values are 2–2.5 times higher compared to cellulose membranes (5–8%) [11]. This decrease in Orange II rejection is likely due to an increase in the number of pores and total free volume in the membrane. A slightly different picture is observed for Remazol: an increase in the terpolymer content in the cellulose matrix to 30 wt.% is accompanied by a moderate decrease in rejection. A further increase in PAN in the system by up to 50 wt.% leads to a decrease in rejection values by almost doubling. With an equivalent ratio of components, the rejection of Remazol is about 20%.

To compare the effectiveness of membranes based on cellulose and PAN, one can consider composite membranes based on aluminum alginate, which are proposed to be used to remove substances simulating antibiotics from organic and aqueous media [40], or membranes obtained from a mixture of polyvinyl alcohol (PVA) and alginate crosslinked with glutaraldehyde [41]. For such systems, comparable values are observed both in terms of permeability (4 kg/m^2^·h^1^·bar^1^) and rejection of the anionic dyes Orange II and Remazol (R < 80%). The observed permeability of the resulting composite membranes differs greatly from commercial membranes NF-90, NF-270, and NF-2, etc., by several times [42], and there are still opportunities to increase it in the future by varying the conditions for forming the membrane and introducing composite particles of different natures.

Thus, the inclusion of the additive PAN into cellulose allows the formation of polymeric membranes with a structure and morphology different from cellulose, which makes it suitable for application in nanofiltration and ultrafiltration processes for the separation of organic or aqueous media.

## 4. Conclusions

For the first time, utilization of the method for the preparation of membranes based on cellulose and copolymers of PAN from concentrated solutions in NMMO has been proposed. The phase composition for the mixed solutions remains discrete (incompatible), i.e., cellulose solutions in NMMO and PAN in NMMO coexist separately. The phase composition and morphology completely determine the rheological behavior of the system and are reflected in the structure of the formed composite films. The non-monotonous change in the viscosity with the PAN content indicates the existence of a specific interaction between cellulose and PAN in NMMO solutions. The structure of the membranes varies depending on the concentration of the additive added, which is reflected in a decrease in the structural order of the cellulose matrix. In consequence, there is an increase in the permeability of the permeate through the membrane. Ethanol permeability varied from 2.2 to 6 kg/m^2^·h·bar depending on the PAN content in the system. Composite membranes have the best rejection of 60% for Orange II and 70% for Remazol model substances. Further optimization of the conditions for the membranes’ formation and the PAN copolymer’s composition may allow the achievement of large flux values.

## Figures and Tables

**Table 1:** Mechanical properties of membranes (wet conditions).

Sample	Thickness, μm	Tensile Strength, MPa	Elongation at Break, %
100 wt.% Cellulose	110 ± 4	3.9 ± 0.3	190 ± 9
90 wt.% Cellulose + 10 wt.% PAN	82 ± 3	3.8 ± 0.3	171 + 11
80 wt.% Cellulose + 20 wt.% PAN	93 ± 5	3.5 ± 0.5	165 ± 10
70 wt.% Cellulose + 30 wt.% PAN	85 ± 2	3.1 ± 0.2	94 ± 7
60 wt.% Cellulose + 40 wt.% PAN	84 ± 3	2.7 ± 0.2	67 ± 3
50 wt.% Cellulose + 50 wt.% PAN	92 ± 2	1.8 ± 0.3	39 ± 5

**Table 2:** Adsorption and Permeance of cellulose and composite films.

Sample	A *, wt.%	P **, kg/(m^2^·h·atm)
100 wt.% Cellulose	10 ± 0.1	2 ± 0.1
90 wt.% Cellulose + 10 wt.% PAN	13 ± 0.2	2.2 ± 0.1
80 wt.% Cellulose + 20 wt.% PAN	18 ± 0.4	2.4 ± 0.1
70 wt.% Cellulose + 30 wt.% PAN	31 ± 0.3	3.4 ± 0.2
50 wt.% Cellulose + 50 wt.% PAN	47 ± 0.3	6 ± 0.4

A *—Adsorption (n-Decan, C_10_H_22_), P **—Permeance (EtOH).

## Data Availability

Not applicable.
